# Interindividual Variation in Cardiorespiratory Fitness: A Candidate Gene Study in Han Chinese People

**DOI:** 10.3390/genes11050555

**Published:** 2020-05-15

**Authors:** Gaowa, Juan Del Coso, Zhuangzhuang Gu, Wuyun Gerile, Rui Yang, Roberto Díaz-Peña, Pedro L. Valenzuela, Alejandro Lucia, Zihong He

**Affiliations:** 1 Institute of Physical Education, Inner Mongolia Normal University, Huhehaote 010022, China; gaowa@imnu.edu.cn (G.); wygrl@imnu.edu.cn (W.G.); 2 Center for Sport Studies, Rey Juan Carlos University, 28943 Fuenlabrada, Spain; 3 Sport Science School, Beijing Sport University, Beijing 100083, China; 2019112049@bsu.edu.cn; 4 Biology Center, China Institute of Sport Science, Beijing 100061, China; yangrui@ciss.cn; 5 Faculty of Health Sciences, Universidad Autónoma de Chile, Talca 3460000, Chile; roberdp78@gmail.com; 6 Department of Systems Biology, University of Alcalá, 28805 Madrid, Spain; pedrol.valenzuela@edu.uah.es; 7 Faculty of Sport Sciences, European University of Madrid, 28670 Madrid, Spain; alejandro.lucia@universidadeuropea.es; 8 Research Institute Hospital 12 de Octubre (‘imas12’), 28041 Madrid, Spain

**Keywords:** VO_2max_, maximal oxygen uptake, single nucleotide polymorphism, genomics, endurance performance

## Abstract

Cardiorespiratory fitness, as assessed through peak oxygen uptake (VO_2peak_), is a powerful health indicator. We aimed to evaluate the influence of several candidate causal genetic variants on VO_2peak_ level in untrained Han Chinese people. A total of 1009 participants (566 women; age [mean ± SD] 40 ± 14 years, VO_2peak_ 29.9 ± 7.1 mL/kg/min) performed a maximal incremental cycling test for VO_2peak_ determination. Genomic DNA was extracted from peripheral whole blood, and genotyping analysis was performed on 125 gene variants. Using age, sex, and body mass as covariates, and setting a stringent threshold *p*-value of 0.0004, only one single nucleotide polymorphism (SNP), located in the gene encoding angiotensin-converting enzyme (rs4295), was associated with VO_2peak_ (β = 0.87; *p* < 2.9 × 10^−4^). Stepwise multiple regression analysis identified a panel of three SNPs (rs4295 = 1.1%, angiotensin II receptor type 1 rs275652 = 0.6%, and myostatin rs7570532 = 0.5%) that together accounted for 2.2% (*p* = 0.0007) of the interindividual variance in VO_2peak_. Participants carrying six ‘favorable’ alleles had a higher VO_2peak_ (32.3 ± 8.1 mL/kg/min) than those carrying only one favorable allele (24.6 ± 5.2 mL/kg/min, *p* < 0.0001). In summary, VO_2peak_ at the pre-trained state is partly influenced by several polymorphic variations in candidate genes, but they represent a minor portion of the variance.

## 1. Introduction

Cardiorespiratory fitness (CRF) is positively associated with endurance exercise performance [1] and is a strong prognostic factor of morbidity and mortality from all causes and, particularly, from cardiovascular disease (CVD) [2,3]. While both physical activity (PA) and exercise training can modify CRF and are inversely associated with morbidity and mortality rates [4], CRF per se is a much stronger predictor of prognosis in CVD and metabolic disorders [5,6]. The measure of an individual’s peak capacity to perform dynamic aerobic exercise is dependent on the synergistic action of pulmonary, cardiovascular and muscle tissue via a suite of physiological actions that effectively transport and deliver oxygen from the atmosphere to mitochondria in working muscles [7,8]. Accordingly, CRF can be assessed by directly measuring the peak oxygen uptake (VO_2peak_) reached during a graded dynamic exercise test until exhaustion, involving large muscle masses (e.g., running or bicycling), or by indirectly estimating this variable from the peak workload achieved. Nevertheless, direct evaluation of VO_2peak_ is considered the gold standard measure of CRF and, indeed, the American Heart Association recently advocated for the routine assessment of this measure as a clinical vital sign [9].

VO_2peak_ is characterized by a high interindividual variability even in people of the same sex, age and with the same level of PA and exercise training. This variability is believed to be related, at least partly, to heredity. A seminal study by Claude Bouchard and colleagues found comparable VO_2peak_ values in brothers of the same sibship, and the similarities in VO_2peak_ were even greater in dizygotic and monozygotic twins [10]. The authors suggested that the genetic effect on VO_2peak_ reached ~40%. In a similar study of 170 individuals and their offspring (n = 259), it was found that about 50% of the interindividual variance in VO_2peak_ corresponded to heritable factors after adjusting for age, sex, body mass, and body composition [11]. These findings have been replicated in subsequent studies with siblings and twins [12] and, to date, it is commonly accepted that VO_2peak_ is influenced by both genetic (~50–60%) and environmental factors. It has also been reported that twins with similar VO_2peak_ values present with comparable levels of a variety of PA indices [13], suggesting that part of the heritability of VO_2peak_ in twins might be due to the similarity of their PA levels. In fact, in a recent analysis of 123,545 single nucleotide polymorphisms (SNPs), only nine were associated with VO_2peak_ [14]. The authors of this study found that those individuals whose genotype was associated with a high VO_2peak_ value had a lower CVD risk (e.g., less visceral fat or lower total blood cholesterol), but they did not calculate the additive effect that the nine SNPs had on the interindividual variability of VO_2peak_. There is therefore controversy on the influence of genetics on VO_2peak_, which mostly likely stems from the discrepancies between studies conducted on siblings/twins vs those conducted on individuals with no familial connection. In this regard, determining the actual genetic contribution to the interindividual variability in VO_2peak_ would be of major importance to inform how environmental factors—including lifestyle—might contribute to heightened VO_2peak_ values. It is possible that if the influence of genetics on VO_2peak_ is low, exercise training might be a determining factor to enhance ‘innate’ VO_2peak_ even in those less genetically predisposed, with obvious subsequent benefits for cardiovascular health. Indeed, previous research has reported VO_2peak_ increases of up to 44% after strenuous training interventions, which would support a strong influence of environmental factors on CRF [15].

Aerobic/endurance exercise-based training appears to be the most effective way to augment VO_2peak_. Exercise training increases rather than decreases the individual differences seen at baseline VO_2peak_ because the response to training itself shows large variation [16]. A genome-wide association study based on 324,611 SNPs found that only 21 SNPs could explain 48.6% of the change in VO_2peak_ induced by a 20-week exercise training program [17]. Among them, rs6552828, located in the acyl-CoA synthase long-chain member 1 (*ACSL1*) gene, accounted by itself for 6% of the training-induced enhancement in VO2_peak_. In a recent meta-analysis of 35 articles on the genetic influence on VO_2peak_ trainability, a total of 97 genes were associated with this phenotype, although only 13 genetic variants were reproduced by more than two investigations [18].

Knowledge on the genetic influence on baseline VO_2peak_ (i.e., in isolation from training) is mainly based on studies conducted on siblings/twins or in individuals of Caucasian descent, and it remains to be determined whether the genetic variants that might be associated with baseline VO_2peak_ are similar or different in individuals of other ethnicities. Thus, the aim of the present study was to assess the influence of several candidate genetic variants in the interindividual variation of baseline CRF measured as VO_2peak,_ in Han Chinese individuals.

## 2. Materials and Methods

### 2.1. Participants

A total of 1047 participants (56% women) volunteered to participate in the study. The sample was recruited from five cities in China: Beijing, Xi’an, Guangzhou, Shenyang, and Tianjin. Inclusion criteria were the following: male/female aged 18–69 years; being of Chinese (Han) descent and unrelated to the other participants; having no CVD, diabetes or abnormal glucose tolerance, or any other acute or chronic disease; and being untrained (i.e., ≤ 2 sessions/week of ≤ 30min of regular physical exercise in the previous 12 months). One week before the start of the investigation, participants were fully informed of the experimental procedures and signed an informed written consent to participate in the investigation. The study protocol was approved by the Institutional Review Board of the China Institute of Sport Science.

### 2.2. Experimental Design

This is an observational cross-sectional study aimed at determining the genetic influence of target genes on the interindividual variability in VO_2peak_ values in untrained Han Chinese individuals. We selected untrained individuals to avoid any influence of exercise training or planned PA in the analysis.

### 2.3. Experimental Protocol

The day of the first experimental trial, participants underwent a medical examination (including medical history and other routine physical examinations) carried out by a licensed physician, to ensure the suitability of all participants to take part in the research protocols. On the same day, whole body dual-energy X-ray absorptiometry (GE Lunar DPX system, Madison, WI, USA) assessments were performed and used to calculate body fat and fat-free mass following previous recommendations [19]. VO_2peak_ (in mL/kg/min) was determined during a continuous incremental exercise test to volitional fatigue performed on a bicycle ergometer (Ergoselect 100, Ergoline GmbH, Bitz, Germany). Before tests, participants were familiarized with the ergometer and with the rating of perceived exertion (RPE), as measured by the Borg 6–20 scale [20]. Participants performed a standardized warm-up (5 min cycling at 20 W and 60 rpm), and the workload (starting at 20 W) was then increased by 25 W (men) or 20 W (women) every 2 minutes until volitional exhaustion. In participants >60 years of age, the workload was increased by 20 W (men) or 15 W (women) every 2 minutes. During the test, gas exchange data were measured ‘breath-by-breath’ with a metabolic cart (MetaMax 3B, Cortex Biophysik GmbH, Leipzig, Germany). Certified calibration gases (16.0% O_2_, 5.0% CO_2_, Cortex Biophysik) and a 3-L syringe were used to calibrate the gas analyzer and the flow meter, respectively, before each test. VO_2peak_ was defined as the highest VO_2_ value (60-s average) obtained during the test. VO_2peak_ was considered valid when participants achieved at least two of the following criteria: (i) RPE >17, (ii) VO_2_ difference between the last two consecutive loads <0.15 L/min, (iii) respiratory exchange ratio >1.1, and (iv) peak heart rate >85% of the age-adjusted estimate [21]. Heart rate was recorded with a chest strap transmitter (Polar RS400, Polar Electro, Kempele, Finland). The environmental temperature was similar in all measurement centers (~22 °C, 40% relative humidity).

On a different day during the week of testing, genomic DNA was extracted from peripheral whole blood samples using the Wizard Genomic DNA Purification Kit (Promega; Madison, WI, USA). Genotyping was performed at Shanghai Benegene Biotechnology, LTD (Shanghai, China). For analysis, a list of 125 SNPs (Table A1 and Table A2, Table A3 and Table A4) for the Han population of Beijing, China (CHB) was obtained from the International HapMap Project database. Haplotype-tag SNPs were selected using the following criteria: minor allele frequency ≥0.01 and measure of linkage disequilibrium (r^2^ > 0.8). Initially, genes associated with cardiovascular responses to exercise were chosen, and genes associated with endurance performance, muscle performance, or body composition were then added as all of these factors might contribute to the value of VO_2peak_ (Table 1).

For high-throughput genotyping of SNPs, we used a matrix-assisted laser desorption/ionization time-of-flight mass spectrometry (MALDI-TOF MS) platform (Agena, San Diego, CA, USA). Primers for the polymerase chain reaction (PCR) and single-base extension were designed using the Assay Designer software package (Assay Design Suite V2.0, Agena, San Diego, CA, USA). Genotyping was performed as described elsewhere [50].

### 2.4. Statistical Analysis

All statistical analyses were performed using SAS 9.4 statistical package (SAS institute, Inc., Cary, NC, USA) and PLINK (v1.07). Hardy–Weinberg Equilibrium (HWE) was tested using *χ*^2^ tests. Linear regression analyses were conducted to assess the association—expressed as standardized regression coefficients (β)—between each SNP and VO_2peak_, with age, body mass and sex as covariates. The Bonferroni correction for multiple comparisons was applied to test for statistically significant associations between SNPs and VO_2peak_, thereby setting the minimum level of significance at *p* < 0.0004 (i.e., 0.05 divided by the number of SNPs, i.e., 125). A multivariable regression analysis was then conducted to assess the overall contribution of the most significant SNPs to the interindividual variability of VO_2peak_. All SNPs with *p* < 0.05 were included, and a regression model with backward elimination was used to filter-out redundant SNPs. By using a threshold of 5.0 points in the variance inflation factor, we avoided multicollinearity. SNPs that were retained in the final backward elimination model were then analyzed with a multivariate regression model using forward selection. The produced regression equation was accepted at a significance level of *p* <0.01. The values of R^2^ were adjusted for the number of cases and parameters in the analysis. The relative contribution (R^2^) of each SNP in relation to the explained variance in VO_2peak_ was calculated as follows (Equation (1)):

Partial contribution (R^2^ adjusted) = ([β for parameter] / Σ [of all β in equation]), (1)

In the SNPs retained in multiple regression, VO_2peak_ values were compared among genotypes by using one-way analysis of variance (ANOVA). When the ANOVA showed a significant F value, pairwise differences were assessed using the Tukey post-hoc test. By using the SNPs retained in multiple regression analyses, we calculated a weighted genotype score to assess the combined influence of the SNPs on VO_2peak_ following the procedure of Williams and Folland [51]. First, each genotype was scored within each SNP by assigning 0 arbitrary units (a.u.) to homozygotes for the allele theoretically associated with low VO_2peak_, 1 a.u. to heterozygotes, and 2 a.u. to homozygotes for the allele associated with high VO_2peak_, following an additive model. Each SNP was then weighted by its β-coefficient (allele effect) based on the assumption that all SNPs of interest have independent effects and contribute in an additive manner to VO_2peak_. Finally, the scores obtained for each SNP were summed to obtain a unique weighted genotype score for each participant (theoretical range: 0–6 a.u.). For clarity, we merged data of participants by using intervals of 1 a.u. Differences in VO_2peak_ between participants in the different groups were assessed by one-way analysis of variance and using the least significant difference post hoc test. Finally, the ability of weighted genotype score to distinguish individuals with low or intermediate CRF (i.e., below or above 28 mL/kg/min, as proposed by Kodama et al. [52]) was assessed using a receiver operating characteristic (ROC) curve and by determining the area under the ROC curve (AUC).

## 3. Results

Of the initial 1047 individuals recruited, valid VO_2peak_ measurements were obtained for 1009 individuals (566 women), and thus only these participants were included in the analyses. The main characteristics of the participants are shown in Table 2.

**Figure 1:**
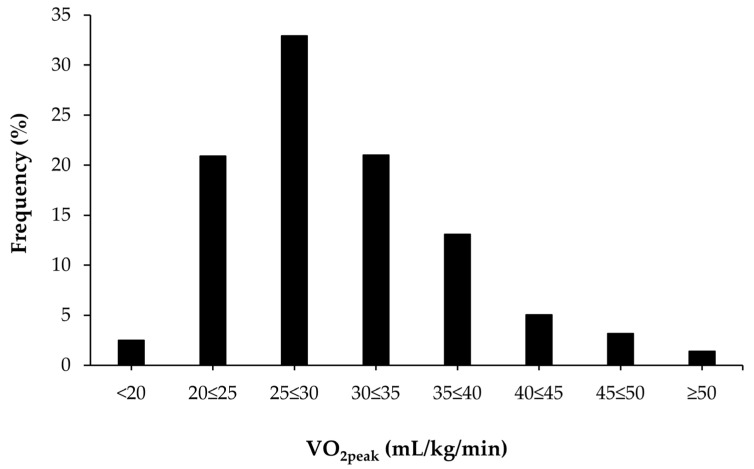
Distribution of peak oxygen uptake (VO_2peak_) data in the study participants.

Figure 1 shows the distribution of VO_2peak_ values in the study sample. Approximately 2.5% of all participants had a VO_2peak_ <20 mL/kg/min and 1.4% had a VO_2peak_ level >50 mL/kg/min.

Genotyping was successful (i.e., successful determinations for all SNPs) in 1006 of 1009 participants (99.7%). From the 125 SNPs analyzed, 10 were discarded because they deviated from HWE (Table A2), 10 because they had a MAF <5% (Table A3), and two because only one genotype was detected across the group of participants (Table A4). From the remaining pool of 103 SNPs, only rs4295, located in the angiotensin-converting enzyme (*ACE*) gene, was significantly associated with VO_2peak_ (*p* < 2.9 × 10^−4^, β = 0.87; minor allele (G) frequency, 38.1%, heterozygosity frequency, 47.4%). 

**Figure 2:**
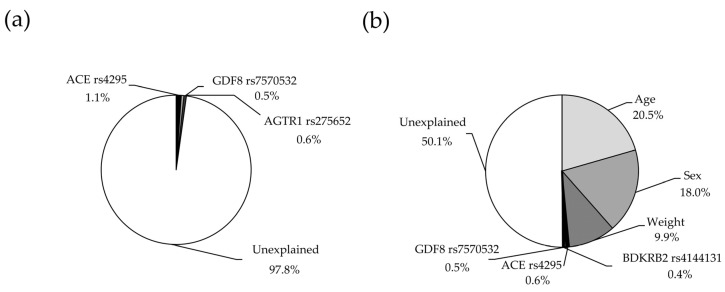
Variance in peak oxygen uptake in the study participants explained by genetic variants alone (**a**) and by genetic variants plus anthropometric covariates (**b**). Abbreviations for gene names: *ACE*, angiotensin-converting enzyme; *AGTR1*, angiotensin II receptor type 1; *BDKRB2*, bradykinin receptor B2; *GDF8*, growth differentiation factor 8 (also known as ‘myostatin’).

In multiple regression analysis, and after excluding those SNPs with collinearity, only three were retained in the final model (*ACE* rs4295, *AGTR1* rs275652, *GDF8* rs7570532), which explained together 2.2% (*p* = 0.0007) of the variance in VO_2peak_ (Figure 2a, statistical power = 0.987). The partial contribution of each SNP to the variance in VO_2peak_ is shown in Table 3. The explained variance of VO_2peak_ increased to 50.1% (*p* < 0.0001) when including covariates such as age, sex and weight in the model (Figure 2b, statistical power = 1.00). 

**Figure 3:**
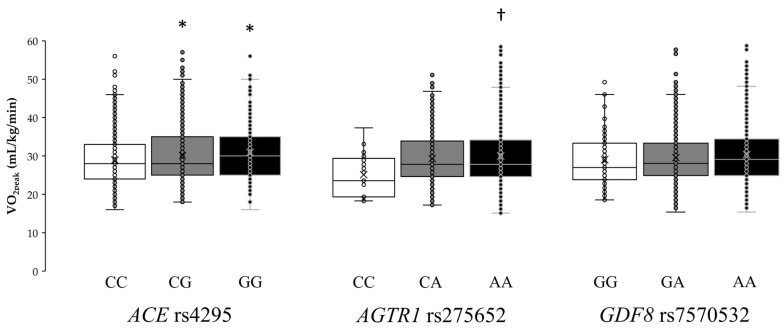
Box-and-whisker plots showing peak oxygen uptake (VO_2peak_) in the study participants according to genetic variations in the genes for angiotensin-converting enzyme (*ACE*; rs4295)*,* angiotensin II receptor type 1 (*AGTR1*; rs275652), and growth differentiation factor 8 (*GDF8,* also known as ‘myostatin’; rs7570532). The lines in the box represent the first, second (median) and third quartiles, and the whiskers represent 1.5 × interquartile ranges. Each dot represents one individual within the specified genotype. (*) Depicts a statistically significant difference from CC genotype in *ACE* rs4295 polymorphism at *p* < 0.05. (†) Depicts a statistically significant difference from CC genotype in *AGTR1* rs275652 polymorphism at *p* < 0.05.

Individual VO_2peak_ values for each genotype of the *ACE* rs4295, *AGTR1* rs275652, and *GDF8* rs7570532 polymorphisms are shown in Figure 3. The one-way ANOVA revealed statistically significant differences in *ACE* rs4295 (F = 4.95, *p* = 0.007) and *AGTR1* rs275652 (F = 3.90, *p* = 0.021) polymorphisms, while the ANOVA did not show differences for *GDF8* rs7570532 (F = 1.64, *p* = 0.194) polymorphism. Specifically, GG homozygotes in *ACE* rs4295 had a mean VO_2peak_ of 31.1 ± 7.9 mL/kg/min, which was higher than that found in heterozygotes (GC, 29.8±6.9 mL/kg/min; *p* = 0.049) or in homozygotes for the common allele (CC, 28.9 ± 6.7 mL/kg/min; *p* = 0.013). In addition, AA homozygotes in *AGTR1* rs275652 had a mean VO_2peak_ of 30.0 ± 7.3 mL/kg/min, which was higher than that found in homozygotes for the minor allele (CC, 25.5 ± 5.3 mL/kg/min; *p* = 0.024).

**Figure 4:**
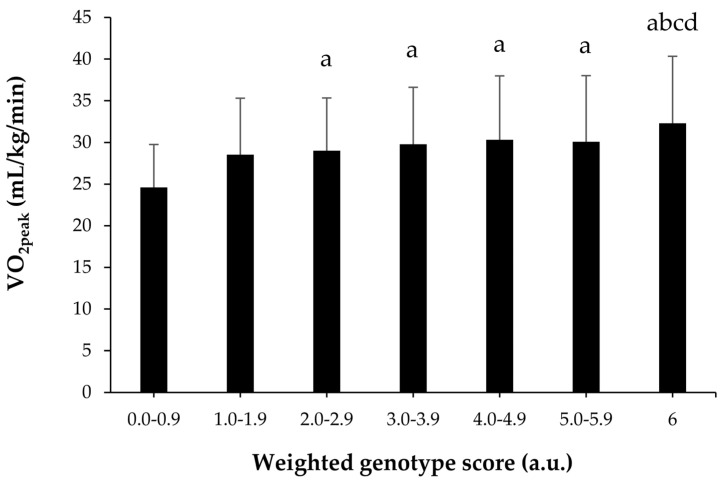
Peak oxygen uptake (VO_2peak_) levels in the study participants according to the genotype score (computed by using a weighted score of angiotensin-converting enzyme rs4295, angiotensin II receptor type 1 rs275652 and growth differentiation factor 8 rs7570532 genotypes). Abbreviations/symbols: a.u., arbitrary units; a, difference from 0–0.9 a.u. at *p*<0.05; b, difference from 1.0–1.9 a.u. at *p* < 0.05; c, difference from 2.0–2.9 a.u. at *p* < 0.05; d, difference from 3.0–3.9 a.u. at *p* < 0.05.

A weighted genotype score was constructed using the three SNPs shown in model 1 of genetic-only influence. Participants were categorized with a genotype score from 0 a.u., indicating the presence of homozygosity for all the alleles associated with a lower VO_2peak_ in *ACE* (rs4295), *AGTR1* (rs275652) and *GDF8* (rs7570532), to 6 a.u., indicating the presence of homozygosity for all the alleles associated with a higher VO_2peak_ in the aforementioned SNPs. A linear effect was found for genotype score on VO_2peak_ (Figure 4). Specifically, the individuals with 6 a.u. had a higher VO_2peak_ than those with scores up to 4.0 a.u. (*p* < 0.05). In addition, participants with scores >2 a.u. had a higher VO_2peak_ than those with scores <1.0 a.u. (*p* < 0.05). ROC analysis showed significant discriminatory accuracy of the weighted genotype score in the identification of individuals with low/intermediate CRF (AUC = 0.542) with a sensitivity of 0.733 and a specificity of 0.305.

## 4. Discussion

CRF, particularly when objectively determined as VO_2peak_, is strongly associated with endurance performance and health outcomes. Indeed, VO_2peak_ reflects the peak integrative ability of the organism to deliver oxygen from the atmosphere to the mitochondria of working muscles. The VO_2peak_ is thus determined, among other factors, by peak cardiac output and pulmonary ventilation, lung diffusion capacity, blood and plasma volume, hemoglobin mass, and muscle capillary density and oxidative capacity [53]. Importantly, the mean values of VO_2peak_ of our participants (29.9 ± 7.1 mL/kg/min or 8.5 metabolic equivalents, i.e., METs) were barely above the minimum healthy threshold for all-cause and CVD mortality in middle-aged men/women (i.e., 8 METs [52]). It is thus of medical importance to determine whether genetic factors (including specific gene variants) are associated with variability of CRF around (i.e., above vs below) the 8-MET cutoff. Previous research in siblings/twins suggests that 50%–60% of the variance of VO_2peak_ is associated with heredity [10,12]. These values seem surprisingly high given the variety of physiological processes and body tissues involved in the uptake and utilization of oxygen in muscle mitochondria. Indeed, there is open debate about the limits of the evidence that support the relative influence of genetics on the variability and trainability of CRF [54,55].

Our findings question the high heritability of VO_2peak_, at least in Chinese individuals with no familial connection. From the 125 SNPs selected for our study, only one (*ACE* rs4295) was associated with VO_2peak_. Also, the best model obtained through multiple regression analyses could only explain ~2.2% of the interindividual variance in VO_2peak_. As in the study by Bye et al. [14], we created a polygenic score to determine whether those individuals with a higher number of alleles associated with VO_2peak_ did indeed present with higher values of this parameter. The only differences found between our findings and those of the Bye et al. study were the number of SNPs included in the polygenic score (7 vs 3, respectively) and the use of an intermediate genotype score for heterozygotes, which was not included by Bye et al. Interestingly, in both studies, participants with the theoretically lowest (or ‘less favorable’) genotype scores had the lowest VO_2peak_ (22–24 mL/kg/min), which was significantly lower than for those with the theoretically highest (or ‘most favorable’) genotype score (~32 mL/kg/min). These findings suggest that only a small number of SNPs are associated with the odds of having high VO_2peak_ values in untrained individuals. The variance of the interindividual variability in VO_2peak_ explained with these genotypes is low and the addition of favorable alleles might produce a change of 8–10 mL/kg/min. This genetic influence might be considerable in clinical terms because each 1-MET (or 3.5 mL/kg/min) increase in CRF has been shown to confer a 12% improvement in survival in Caucasian (North-American) men [6]. Moreover, as mentioned above, it is of clinical importance to surpass the 8-MET threshold, and in fact, adults with a CRF clearly above this level (>10 METs) have a remarkably reduced CVD risk [56]. In this regard, the probability of surpassing the 8-MET threshold (equivalent to 28 mL/kg/min) was doubled in those participants that carried the six ‘favorable’ alleles (Figure 4).

Only three SNPs were included in the final multiple regression model. *ACE* rs4295 has not been previously associated with endurance performance, but it is located in the same linkage disequilibrium block as the widely studied *ACE* insert(I)/deletion(D) polymorphism (rs4340) [57]. The *ACE* gene encodes angiotensin-converting enzyme and the I allele might be associated with lower circulating levels of enzyme, and the II genotype potentially associated with performance in endurance athletes (odds ratio 1.35; 95% confidence interval, 1.17 to 1.55 [58]). However, several studies have found no association between the *ACE* I/D genotype and VO_2peak_ values in trained [59] and untrained [60,61] individuals. With regard to the *ACE* rs4295 variation found in the present study, although its influence on CRF needs to be replicated in other cohorts, our findings bolster the role of angiotensin-converting enzyme and its coding gene as predictors of CRF-related phenotypes. We also found that carriage of the C allele in the *AGTR1* rs275652 polymorphism was negatively associated with VO_2peak_ values. This gene encodes the angiotensin II receptor 1 (AT_1_R), and polymorphisms in *AGTR1* have been suggested to be involved in the physiological response to hypoxia [62]. AT_1_R is broadly expressed in different tissues and mediates most of the classical actions of angiotensin II, including vasoconstriction and vascular smooth muscle cell proliferation [63]. Thus, under hypoxic conditions, angiotensin II engages AT_1_R to modulate the pulmonary vasoconstrictive response [64]. Although speculative, it is possible that the C allele in *AGTR1* rs275652 might exacerbate pulmonary vasoconstriction during exercise owing to a higher activation of AT_1_R for a given concentration of angiotensin II [65]. The last SNP included in the model explaining VO_2peak_ was rs7570532, a genetic variation in *GDF8* encoding myostatin. This and other SNPs in *GDF8* have been indirectly associated with a major cardiometabolic condition, obesity [39], but other authors have reported no association of rs7570532 with endurance performance [40]. Myostatin controls the differentiation and proliferation of skeletal muscle throughout embryonic development and regulates muscle growth during adulthood. Mutations in *GDF8* that produce non-functional myostatin result in the increased growth of skeletal muscle, demonstrating the existence of a powerful mechanism to control muscle size in normal individuals through this protein [66]. Based on these findings and given the positive association between muscle mass and VO_2peak_ [66,67,68], it is possible that *GDF8* rs7570532 confers a small but significant predisposition to higher VO_2peak_ values. Further research on these three SNPs is clearly warranted.

We acknowledge that the current investigation has some limitations. First, our study sample was heterogeneous in terms of age, sex, and anthropometric characteristics (Table 1). Although we used these variables as covariates in linear regression analyses, the high variability of these variables might have partially influenced our results. In fact, when they were included in multiple regression analyses (Figure 2b), the explained variance of VO_2peak_ increased up to 50.1%. Second, our study only included participants of Han Chinese descent and the results might therefore not be applicable to other ethnicities. Of note, the Han Chinese constitute the world’s largest ethnic group (constituting ~18% of the global population), but further studies in other large ethnic groups will be needed to confirm/discard the generalizability of these results. Lastly, we only analyzed 125 SNPs and thus it is plausible that other candidate genes might have an influence on VO_2peak_. 

## 5. Conclusions

The present study shows that in a cohort of untrained Han Chinese individuals, VO_2peak_ is influenced by a very few polymorphic variations in key genes even in isolation of training adaptations. The genetic influence accounted for ~2.2% of the interindividual variance in VO_2peak_, at least with the 125 SNPs included in this investigation. Although more research is needed, these data suggest that environment, probably more than genetics, is responsible for most of the interindividual variability in VO_2peak_ among healthy Han Chinese adults.

## Figures and Tables

**Table 1:** Target genes selected for the investigation.

Gene	Numbers of SNPs	Chromosome Location	References
*ACE*	3	chr17:58,908,166-58,928,711	[22]
*ACE2*	2	chrX:15,489,077-15,529,058	[23,24]
*ACSL1*	15	chr4: 185,911,544-185,986,209	[17,25]
*ACTN3*	1	chr11:66,313,866-66,330,800	[26,27]
*AGT*	13	chr1:228,902,892-228,918,564	[28,29]
*AGTR1*	9	chr3:149,898,348- 149,943,480	[30,31]
*AGTR2*	3	chrX:115,214,031-115,221,847	[32]
*BDKRB2*	28	chr14:95,738,950-95,782,536	[33]
*FGF21*	2	chr19:53,949,156-53,955,394	[34]
*FGFR2*	1	chr10:123,237,848-123,357,972	[34]
*FNDC5*	3	chr1:33,327,869-33,338,083	[35]
*FST*	3	chr5: 52,812,352-52,817,659	[36,37]
*FTO*	3	chr16:53,737,875-54,155,853	[38]
*GDF8*	4	chr2:190,920,423-190,927,455	[39,40]
*IL-6*	7	chr7:22,733,345-22,738,141	[41]
*IL-15*	2	chr4:142,557,752-142,665,140	[42,43]
*ITLN1*	5	chr1:160,846,329-160,854,960	[44]
*PGC-1α*	6	chr4: 23,756,664-23,905,712	[45]
*PGC* *-1β*	1	chr5:149,109,861-149,234,585	[45]
*PPRC1*	1	chr10: 103, 880, 777-103, 902, 078	[45]
*PRDM16*	2	chr1: 2,985,732-3,355,185	[46]
*PYY*	5	chr17:39,385,633-39,437,363	[47]
*REN*	5	chr1: 202,390,571-202,402,088	[48]
*RETN*	1	chr19:7,639,972-7,641,340	[49]

Abbreviations: SNP, single nucleotide polymorphism. Abbreviations for gene names: *ACE*, angiotensin-converting enzyme; *ACE2*, angiotensin-converting enzyme 2; *ACSL1*, acyl-CoA synthase long-chain member 1; *ACTN3*, alpha-actinin-3; *AGT*, angiotensinogen; *AGTR1*, angiotensin II receptor type 1; *AGTR2*, angiotensin II receptor type 2; *BDKRB2*, bradykinin receptor B2; *FGF21*, Fibroblast growth factor 21; *FGFR2*, fibroblast growth factor receptor 2; *FNDC5*, fibronectin type III domain-containing protein 5; *FST*, follistatin; FTO, fat mass and obesity-associated protein (also known as ‘alpha-ketoglutarate-dependent dioxygenase’; *GDF8*, growth differentiation factor 8 (also known as ‘myostatin’); *IL-6*, interleukin 6; *IL-15*, interleukin 15; *ITLN1*, intelectin 1; PGC-1α, peroxisome proliferator-activated receptor-gamma coactivator (PGC)-1alpha; PGC-1β, peroxisome proliferator-activated receptor-gamma coactivator (PGC)-1beta; peroxisome *PPRC1*, proliferator-activated receptor gamma, coactivator-related 1; *PRDM16*, PR domain containing 16; *PYY*, peptide YY; *REN*, renin; *RETN*, resistin.

**Table 2:** Main characteristics of the study participants (N = 1009) and their association with peak oxygen uptake.

Variable	Mean ± SD	Range	β	*p*-Value
Age (year)	40 ± 14	19–69	−0.27	<0.001
Height (cm)	165.3 ± 8.3	146.2–187.0	0.31	<0.001
Body mass (kg)	64.3 ± 11.6	39–104	−0.01	0.523
Body mass index (kg/m^2^)	23.4 ± 3.1	15.6–34.8	−0.64	<0.001
Body fat (%)	27.1 ± 8.8	4.5–44.5	−0.58	<0.001
Fat-free mass (kg)	43.8 ± 9.5	24.8–70.1	0.31	<0.001

**Table 3:** List of single nucleotide polymorphisms associated with peak oxygen uptake in the study participants. Model 1, with genetic-only influence; model 2 with covariates.

	SNP	Partial R^2^	*p*-Value
**Model 1**	*ACE* rs4295	0.0110	0.0024
	*AGTR1* rs275652	0.0056	0.0293
	*GDF8* rs7570532	0.0053	0.0342
**Model 2**	Age	0.2052	<0.0001
	Sex	0.1800	<0.0001
	Weight	0.0994	<0.0001
	*ACE* rs4295	0.0063	0.0015
	*GDF8* rs7570532	0.0046	0.0058
	*BDKRB2 rs4144131*	0.0037	0.0135

Abbreviation: SNP, single nucleotide polymorphism; Abbreviations for gene names: *ACE*, angiotensin-converting enzyme; *AGTR1*, angiotensin II receptor type 1; *BDKRB2*, bradykinin receptor B2; *GDF8*, growth differentiation factor 8 (also known as ‘myostatin’).

## Appendix A

**Table A1:** List of SNPs investigated for association with VO_2peak_ (mL/kg/min) in Han Chinese untrained individuals.

Gene	SNP	MA	MAF
*ACE*	rs4295	G	38.1
*ACE*	rs4341	G	34.9
*ACE*	rs4363	G	38.7
*ACE2*	rs6632677	C	9.2
*ACSL1*	rs10022018	G	24.4
*ACSL1*	rs11732302	C	24.7
*ACSL1*	rs12503643	G	45.9
*ACSL1*	rs12644905	T	19.6
*ACSL1*	rs13126272	T	11.0
*ACSL1*	rs1803898	A	7.3
*ACSL1*	rs2292898	C	7.8
*ACSL1*	rs13120078	A	8.2
*ACSL1*	rs2280297	C	47.6
*ACSL1*	rs2292899	A	38.1
*ACSL1*	rs3749233	A	25.9
*ACSL1*	rs3792312	G	41.7
*ACSL1*	rs4069938	G	37.7
*ACSL1*	rs6552828	G	35.2
*ACSL1*	rs902177	C	28.2
*ACTN3*	rs1815739	T	41.7
*AGT*	rs10864770	T	34.5
*AGT*	rs11568046	C	12.9
*AGT*	rs2478523	C	46.0
*AGT*	rs2478544	C	21.8
*AGT*	rs2493132	T	37.3
*AGT*	rs3789671	G	45.5
*AGT*	rs3789678	T	18.7
*AGT*	rs3889728	A	49.4
*AGT*	rs5050	G	14.8
*AGT*	rs6687360	C	33.1
*AGT*	rs699	T	19.7
*AGT*	rs7079	A	16.2
*AGT*	rs7536290	G	22.0
*AGTR1*	rs2131127	T	37.4
*AGTR1*	rs275652	C	13.6
*AGTR1*	rs3772616	A	17.8
*AGTR1*	rs385338	G	18.0
*AGTR1*	rs5182	C	28.1
*AGTR1*	rs6801836	C	14.2
*BDKRB2*	rs10130005	C	18.3
*BDKRB2*	rs10132462	T	28.1
*BDKRB2*	rs11160322	C	23.0
*BDKRB2*	rs11627176	G	12.0
*BDKRB2*	rs11627761	T	15.3
*BDKRB2*	rs11848502	T	30.1
*BDKRB2*	rs12433275	T	16.3
*BDKRB2*	rs12888402	C	16.7
*BDKRB2*	rs1799722	C	48.0
*BDKRB2*	rs1959053	T	25.3
*BDKRB2*	rs2069575	A	20.4
*BDKRB2*	rs2069578	G	39.1
*BDKRB2*	rs2069586	A	16.5
*BDKRB2*	rs2069588	T	18.0
*BDKRB2*	rs2369521	G	35.6
*BDKRB2*	rs4144131	A	43.9
*BDKRB2*	rs4900315	C	46.5
*BDKRB2*	rs4900318	A	49.7
*BDKRB2*	rs4905470	A	20.0
*BDKRB2*	rs4905474	A	37.1
*BDKRB2*	rs6575577	G	22.5
*BDKRB2*	rs7155797	T	44.4
*BDKRB2*	rs7161665	C	47.9
*BDKRB2*	rs8013400	T	28.5
*BDKRB2*	rs8016905	A	32.7
*BDKRB2*	rs885818	T	13.4
*BDKRB2*	rs945039	T	42.9
*FGFR2*	rs2071616	T	8.8
*FNDC5*	rs16835198	T	47.7
*FNDC5*	rs3480	G	24.8
*FST*	rs3797296	G	17.3
*FST*	rs3797297	T	12.8
*FTO*	rs1421085	C	10.4
*FTO*	rs1558902	A	10.6
*FTO*	rs9939609	A	10.4
*GDF-8*	rs16832288	A	19.9
*GDF-8*	rs7570532	G	26.0
*IL-15*	rs1057972	A	49.9
*IL-6*	rs1524107	C	29.3
*IL-6*	rs2069840	G	7.3
*IL-6*	rs2069830	G	27.2
*IL-6*	rs2069837	G	20.1
*IL-6*	rs2069852	G	37.0
*ITLN1*	rs2274906	A	36.5
*ITLN1*	rs2274910	T	29.6
*ITLN1*	rs2297560	T	13.9
*ITLN1*	rs6427552	C	24.7
*PGC-1α*	rs12374310	C	43.9
*PGC-1α*	rs12650562	C	49.5
*PGC-1α*	rs251468	T	19.8
*PGC-1α*	rs4452416	G	13.4
*PGC-1α*	rs4697425	G	30.8
*PGC-1α*	rs6821591	C	29.9
*PRDM16*	rs12409277	C	42.0
*PRDM16*	rs2236518	A	44.5
*PYY*	rs10853114	C	37.8
PYY	rs12953033	A	6.9
*PYY*	rs162430	G	35.3
*PYY*	rs1859223	G	27.2
*REN*	rs11571078	T	12.7
*REN*	rs1464816	T	24.4
*REN*	rs2368564	T	20.3
*REN*	rs4951313	G	29.1
*REN*	rs5707	G	40.3
*RETN*	rs3745367	A	35.5

Abbreviations: MA, minor allele; MAF, minor allele frequency; SNP, single nucleotide polymorphism; VO_2peak_, peak oxygen uptake. Abbreviations for gene names: *ACE*, angiotensin-converting enzyme; *ACE2*, angiotensin-converting enzyme 2; *ACSL1*, acyl-CoA synthase long-chain member 1; *ACTN3*, alpha-actinin-3; *AGT*, angiotensinogen; *AGTR1*, angiotensin II receptor type 1; *AGTR2*, angiotensin II receptor type 2; *BDKRB2*, bradykinin receptor B2; *FGF21*, Fibroblast growth factor 21; *FGFR2*, fibroblast growth factor receptor 2; *FNDC5*, fibronectin type III domain-containing protein 5; *FST*, follistatin; FTO, fat mass and obesity-associated protein (also known as ‘alpha-ketoglutarate-dependent dioxygenase’; *GDF8*, growth differentiation factor 8 (also known as ‘myostatin’); *IL-6*, interleukin 6; *IL-15*, interleukin 15; *ITLN1*, intelectin 1; PGC-1α, peroxisome proliferator-activated receptor-gamma coactivator (PGC)-1alpha; *PRDM16*, PR domain containing 16; *PYY*, peptide YY; *REN*, renin; *RETN*, resistin.

## Appendix B

**Table A2:** List of SNPs discarded for analyses because did not meet the Hardy–Weinberg equilibrium.

Gene	SNP	MA	MAF
*ACE2*	rs2074192	T	42.6
*ACE2*	rs6632677	C	9.2
*AGTR1*	rs12721241	A	12.0
*AGTR1*	rs2675511	G	13.9
*AGTR2*	rs5193	T	15.9
*AGTR2*	rs12840631	G	18.1
*AGTR2*	rs6608590	T	41.6
*BDKRB2*	rs4900313	A	16.2
*PGC1β*	rs17110586	G	14.8
*PRC*	rs17114388	G	19.7

Abbreviations: see Table A1.

**Table A3:** List of SNPs discarded for analyses because the frequency of the minor allele was inferior to 5%.

Gene	SNP	MA	MAF
*FGF21*	rs838133	A	1.1
*FGF21*	rs838145	G	1.3
*FNDC5*	rs726344	A	0.2
*FST*	rs12152850	T	1.6
*GDF-8*	rs1805086	C	0.2
*GDF-8*	rs3791784	G	2.3
*IL-6*	rs1800795	C	0.7
*IL-15*	rs1589241	T	0.8
*IL-6*	rs1554606	T	1.8
*ITLN1*	rs11265509	T	4.7

Abbreviations: see Table A1.

**Table A4:** List of SNPs discarded for analyses because all individuals of the sample had the same genotype.

Gene	SNP	Genotype
*AGTR1*	rs12721276	CC
*PYY*	rs432747	GG

Abbreviations: see Table A1.
